# Association Between Cytological and Histopathological Diagnoses of Neoplastic and Non-Neoplastic Lesions in Oral Cavity from Dogs and Cats: An Observational Retrospective Study of 103 Cases

**DOI:** 10.3390/vetsci12020075

**Published:** 2025-01-21

**Authors:** Paula Brilhante-Simões, Leonor Delgado, Ângela Martins, Augusto Silva, Luís Monteiro, Ricardo Marcos, Justina Prada

**Affiliations:** 1 INNO Veterinary Laboratories, R. Cândido de Sousa 15, 4710-300 Braga, Portugal; paulabrilhante@inno.pt (P.B.-S.); augustosilva@inno.pt (A.S.); 2 Animal and Veterinary Sciences Department, University Institute of Health Sciences (IUCS), Advanced Polytechnic and University Cooperative, CESPU CRL, 1317, 4585-116 Gandra, Portugal; 3 Department of Veterinary Sciences, University of Trás-os-Montes and Alto Douro, 5000-801 Vila Real, Portugal; angela@utad.pt (Â.M.); jprada@utad.pt (J.P.); 4 GIPOC—Comparative Oral Pathology Research Group, University Institute of Health Sciences—Advanced Polytechnic and University Cooperative (IUCS-CESPU), 4585-116 Gandra, Portugal; luis.monteiro@iucs.cespu.pt; 5 UNIPRO—Oral Pathology and Rehabilitation Research Unit, University Institute of Health Sciences—Advanced Polytechnic and University Cooperative (IUCS-CESPU), 4585-116 Gandra, Portugal; 6 Animal and Veterinary Research Centre (CECAV), Associate Laboratory for Animal and Veterinary Sciences (AL4AnimalS), University of Trás-os-Montes and Alto Douro, 5000-801 Vila Real, Portugal; 7 Cytology and Hematology Diagnostic Services, Laboratory of Histology and Embryology, Department of Microscopy, ICBAS-School of Medicine and Biomedical Sciences, University of Porto (U.Porto), Rua de Jorge Viterbo Ferreira, 228, 4050-313 Porto, Portugal; rmarcos@icbas.up.pt

**Keywords:** cytology, oral lesions, neoplasia, canine, feline

## Abstract

Cytology is an exam frequently used as a diagnostic technique to evaluate animal oral lesions. However, the retrospective studies published comparing cytology and histology in oral pathology are scarce. This study aimed to determining the association between diagnostic cytology and histopathology in neoplastic and non-neoplastic samples from oral cavity lesions of dogs and cats. Cytological and histological samples (*n* = 103) of oral lesions from 70 dogs and 33 cats collected over 12 years were compared in terms of agreement, considering the histopathology as the gold standard. Of 103 specimens, 78 were classified as neoplastic, and 25 were non-neoplastic. The sensitivity, specificity, and accuracy of cytology in neoplastic and non-neoplastic lesions were 84.6%, 96.0%, and 87.4%. Of the overall 103 cases, 75.7% (*n* = 78) were neoplastic and revealed an agreement that was complete in 64.1% (*n* = 50) and partial in 17.9% (*n* = 14) samples, and the remaining 17.9% (*n* = 14) revealed disagreement; regarding the non-neoplastic diagnoses (24.3%, *n* = 25) the agreement was complete in 68.0% (*n* = 17), partial for 20.0% (*n* = 5) samples, and the remaining 12.0% (*n* = 3) revealed disagreement. A statistical association between neoplastic cases and agreement was obtained (*p* < 0.0001). The high agreement verified between the cytological and histopathological diagnoses emphasizes the role of cytology as the first approach method to an oral lesion in dogs and cats.

## Simple Summary

Diagnosing oral lesions in dogs and cats is crucial for effective treatment, as these lesions may range from benign conditions to malignant cancers. This study analyzed 103 cases of oral lesions, comparing cytology, a fast minimally invasive diagnostic method, with histopathology, the gold standard for diagnosis. Cytology demonstrated high accuracy (87.4%) in distinguishing cancerous from non-cancerous lesions and showed complete agreement with histopathology in most cases (65%). However, some discrepancies arose, often due to challenges such as an inadequate sample quality or the inability of cytology to assess tissue architecture. Despite these limitations, cytology proved to be an invaluable first diagnostic step, offering veterinarians a rapid and cost-effective tool to guide early treatment decisions. By identifying potential cancers at an earlier stage, cytology contributes to improve outcomes for pets. The findings are valuable to society by providing a rapid and affordable first step in diagnosing oral lesions, leading to better outcomes for pets.

## 1. Introduction

The cytology of these lesions may serve as a first diagnostic approach for classifying oral cavity lesions [1,2]. It is a rapid, inexpensive, and minimally invasive procedure whether performed by fine needle with or without aspiration or by impression [1,2,3,4].

The procedure can be performed using inexpensive equipment [5,6], is generally easy to execute, and allows for significantly faster sample preparation compared to histopathology, enabling immediate examination of the collected tissue samples [5]. However, due to the absence of tissue architecture in cytological samples, accurately categorizing lesions can be quite challenging [7]. In practical terms, cytology can swiftly facilitate the assessment and classification of lesions as neoplastic or non-neoplastic in a substantial proportion of samples [8,9,10,11,12].

The presence of masses in the oral cavity is frequently noted in dogs and cats, including benign and malignant neoplastic lesions, as well as non-neoplastic lesions [13,14] with diverse origins and varied characteristics [15,16].

Oral tumor lesions in small animals account for 6–7% of all tumors in dogs and 3% of all tumors in cats [17]. In this location, the most common tumors diagnosed in dogs are melanomas [2,15,18], while in cats, squamous cell carcinomas are the most common (SCC) [2,19,20].

Several studies showed an association between cytology and histology in dogs and cats in many locations [21], such as lymph nodes [22], cutaneous and subcutaneous lesions [1,23], spleen [24], or specific lesions such as thymomas [25], or even studies on tumors in dogs [26] or with specific lesions such as mammary tumors [27,28,29,30], bone lesions, and osteosarcomas [31] or angiosarcomas [32]. So far, only a single study has correlated cytology and histopathology in oral lesions [2]. In humans, several studies have addressed this association between cytology and histopathology in oral lesions [33,34,35,36,37].

The aim of this retrospective study was to determine the association between diagnostic cytology and histopathology in neoplastic and non-neoplastic samples from oral cavity lesions of dogs and cats submitted by veterinary clinics and hospitals in Portugal, using histological diagnosis as the gold standard.

## 2. Materials and Methods

This was an observational retrospective study of oral lesion samples of dogs and cats from 21 veterinary hospitals and 55 clinics in Portugal, all submitted to the Pathology Laboratory—INNO, in the period between 2010 and 2022.

Cases were included when the same lesion had both cytological and histopathological specimens and were excluded if one of the specimens was not available or was poorly preserved. With regard to the cytological cases, most cases were collected by fine needle aspiration; however, for some, specific information on the sampling method was not available.

The database was collected from the Clinidata^®^ (Clinidata XXI version 5.3.25, Maxdata Software, S.A., Carregado, Portugal) system and organized with Microsoft Excel^®^ (Microsoft, Redmond, WA, USA). The STROBE guidelines for reporting observational cross-sectional studies were followed [38].

Cytological specimens were stained using Romanowsky stains (Hemacolor, Merck KGaA, Darmstadt, Germany). Histologic specimens were fixed in 10% neutral buffered formalin, routinely processed, and stained using hematoxylin and eosin (H&E) [1,2,22].

Histopathological diagnosis was performed based on the World Health Organization (WHO) histological classification of tumors of the alimentary system of domestic animals [39].

After applying the inclusion and exclusion criteria to a total of 138 cytological reports of dogs and cats, 103 cases remained. The exclusion criteria applied were scarce or absent cellularity, blood contamination, and exposure to formaldehyde. The cases with these criteria were excluded to avoid possible bias.

The variables analyzed were the species, gender, age, breed, location of the lesion, and cytological and histopathological diagnoses.

All cytological and histopathological diagnoses were reviewed independently by two experienced pathologists (PBS and LD, respectively), not aware of the previous cytological or histopathological diagnoses. The discordant cases were reviewed by a third observer (JP) and discussed together to achieve a consensus.

The diagnoses of cytology and histopathology of the oral lesions were compared in terms of agreement, considering the histopathology result as the gold standard [1]. It was considered as complete agreement when both diagnoses matched for cell type and its classification; partial agreement was defined as when the cell type matched, but the classification did not; and when both type and classification did not match, it was deemed as a disagreement [40].

To obtain the sensitivity, specificity, and accuracy of the cytological and histopathological diagnoses in both species, the data were classified as true positive (TP), true negative (TN), false positive (FP), and false negative (FN). TP included samples that were positive for neoplasia detection on both cytology and histology; TN signified the samples were positive for non-neoplasia diagnosis on both cytology and histology; FP signified the samples were positive for neoplasia diagnosis on cytology but negative for neoplasia diagnosis on histology; FN signified the samples were negative for neoplasia diagnosis on cytology but positive for neoplasia diagnosis on histology [1,22,41,42].

Statistical analysis was conducted using JMP 13 2016 SAS Institute, Cary, NC, USA. The chi-square test was used for categorical variables. Statistical significance was established at the level of 0.05 (5%) [34].

## 3. Results

### 3.1. Population Characteristics

A total of 103 animals, 70 (68%) dogs and 33 (32%) cats, were included. In the dog group, 32 (45.7%) were female, and 38 (54.3%) were male, with an age range from 1 to 16 years (mean ages 8.66 ± 4.3 years); in the cat group, 18 (54.5%) were female, and 15 (45.5%) were male, with an age range from 1 to 18 years (mean ages 8.67 ± 4.3 years). The age information was absent for two dogs and three cats.

Of the 103 dogs in our sample, 20 (30.3%) were of undetermined breed. The remaining 83 animals were of the following breeds: Labrador Retriever (*n* = 14; 21.2%), French Bulldog (*n* = 5; 7.6%), Cocker Spaniel, Golden Retriever (*n* = 4; 6.1% each), Boxer and German Shepherd (*n* = 3; 4.5% each), Yorkshire Terrier (*n* = 2; 3.0%) and Beagle, Dogue of Bordeaux, Estrela Mountain Dog, Pekingese, Pinscher, Pitbull Terrier, Portuguese Podengo, Pyrenees Mountain Dog, Rhodesian Ridgeback, Rottweiler, and Samoyed (*n* = 1; 1.5% each).

The most common cat breeds (by descendent order) were: Domestic short-haired (DSH) (*n* = 24; 88.9%), Norwegian Forest, Persian, and Siamese (*n* = 1; 3.7% each). The information about breed was absent for four dogs and six cats, respectively.

Thirty-five (25.4%) samples of the 138 initial samples were excluded because the cytological results were not satisfactory: twenty-eight (80.0%) of these cases presented scarce or absent cytological cellularity and three (8.6%) cases scarce or absent histological cellularity; two (5.7%) cases presented slides exposed to formaldehyde; and two (5.7%) cases were excluded due to blood-contaminated samples.

### 3.2. Oral Lesions—Location

The location of the dogs’ oral lesions were the gingiva and lips (each with 17 cases (24.3%), mouth not otherwise specified (MNOS) (*n* = 15; 21.4%), palate (*n* = 5; 7.1%), lymph nodes and jaw (*n* = 4; 5.7%; each), maxilla, salivary glands and tongue (*n* = 2; 2.9%; each), and oropharynx and tonsils (*n* = 1; 1.4%; each) (Table 1).

The location of the cats’ oral lesions was the tongue (n = 9; 27.3%), MNOS (*n* = 7; 21.2%), gingiva (*n* = 5; 15.2%), lip (*n* = 3; 9.1%), jaw and palate (*n* = 2; 6.1%; each), and dental, maxilla, oropharynx, salivary glands, and tonsils (*n* = 1; 3.0%; each) (Table 1).

For statistical purposes, the locations with less than 10 cases were not considered (lymph node, jaw, dental, maxilla, salivary gland, palate, tonsils, and oropharynx). A chi-square test was performed between the location and level of agreement between the cytological and histological diagnoses (Table 2). A *p*-value of 0.9074 was obtained, where the cases with most agreements were found on the MNOS (*n* = 17; 22.67%), and the cases with the most partial agreements and disagreements were found on the gingiva (*n* = 5; 6.67%; each).

### 3.3. Oral Lesions—Diagnosis

**Figure 1:**
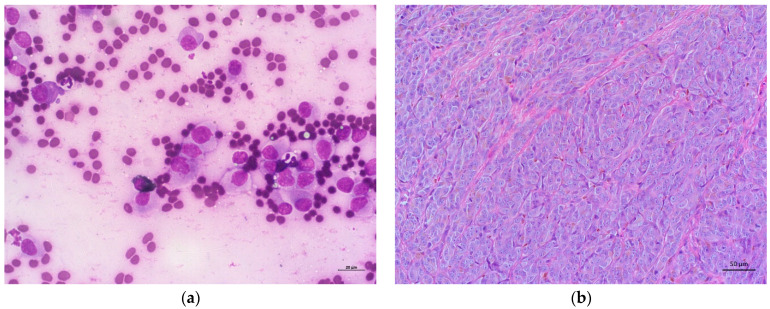
Representation of the cytology (**a**) and histopathology (**b**) of a canine amelanotic oral melanoma. Round to polygonal cells, disposed single or in small clusters, with round to pleomorphic nuclei and a moderate amount of lightly basophilic cytoplasm with rare black granulation, showing mild to moderate anisocytosis and anisokaryosis (**a**). Packets of densely cellularly and infiltrative round to polygonal cells, disposed on a small amount of fibrovascular stroma. The nuclei are round to oval, with a central prominent nucleoli and moderate amount of eosinophilic cytoplasm with poorly distinct cell borders and scarce brown granular pigment (**b**). (**a**)—Hemacolor^®^ 400×; (**b**)—H&E 200×.

**Figure 2:**
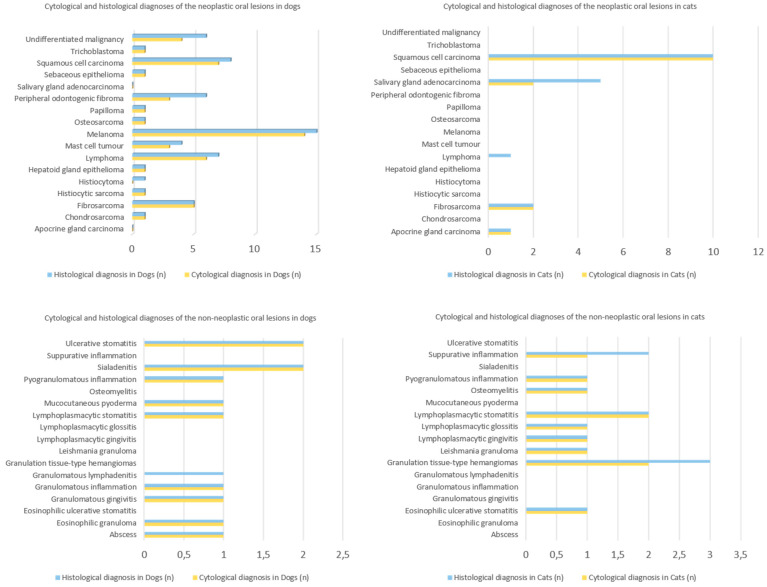
Cytological and histological diagnoses of the neoplastic and non-neoplastic oral lesions in dogs and cats.

When comparing the cytological and histological diagnoses of neoplastic oral lesions in dogs, we obtained the same diagnosis for chondrosarcoma, fibrosarcoma, histiocytic sarcoma, hepatoid gland epithelioma, osteosarcoma, papilloma, sebaceous epithelioma, and trichoblastoma. For the remaining neoplasms, we obtained a lower number of cytological diagnoses compared to the histological diagnoses: histiocytoma, lymphoma, mast cell tumor, melanoma (Figure 1a,b), peripheral odontogenic fibroma, squamous cell carcinoma, and undifferentiated malignancy. The values obtained for the previous data are shown in Figure 2.

**Figure 3:**
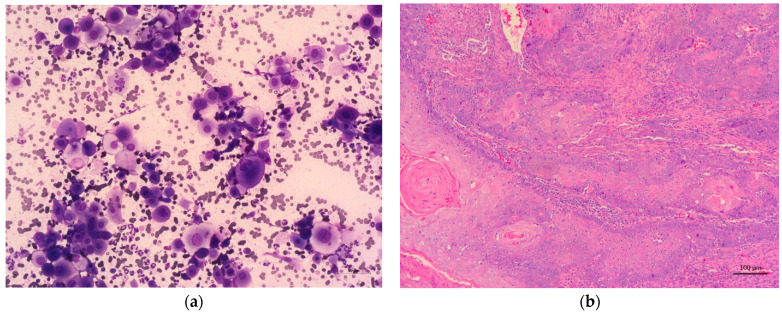
Representation of the cytology (**a**) and histopathology (**b**) of a feline oral squamous cell carcinoma. Epithelial cells with large atypical nuclei, moderate to abundant and variable basophilic cytoplasm, disposed in small clusters, showing nuclear–cytoplasmic asynchrony and moderate to marked anisocytosis and anisokaryosis. Notice the presence of small groups of non-degenerated neutrophils and red blood cells (**a**); Nests and cords of epithelial infiltrative neoplastic cell, with a variable amount of squamous cell differentiation, sometimes with formation of keratin perls. These cells are disposed on a moderate fibrovascular stroma with mild to moderate lymphocytic infiltration (**b**). (**a**)—Hemacolor^®^ 200×; (**b**)—H&E 100×.

When comparing the cytological and histological diagnoses of neoplastic oral lesions in cats, we obtained the same diagnosis for apocrine gland carcinoma, fibrosarcoma, and squamous cell carcinoma (Figure 3a,b). For the remaining neoplasms, we obtained a lower number of cytological diagnoses compared to the histological diagnoses: lymphoma and salivary gland adenocarcinoma (Figure 2).

**Figure 4:**
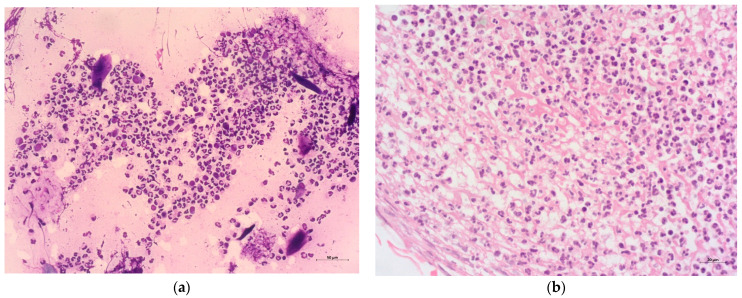
Representation of the cytology (**a**) and histopathology (**b**) of a dog with an ulcerative stomatitis. Abundant inflammatory population composed of innumerous degenerated and non-degenerated neutrophils and fewer lymphocytes and macrophages. Presence of scarce keratinic material and cellular debris (**a**). Large population of degenerated and non-degenerated neutrophils admixed with moderate eosinophilic and filamentous material (fibrin) (**b**). (**a**)—Hemacolor^®^ 200×; (**b**)—H&E 400×.

When comparing the cytological and histological diagnoses of non-neoplastic oral lesions in dogs, we obtained the same diagnosis for abscess, eosinophilic granuloma, granulomatous gingivitis, granulomatous inflammation, lymphoplasmacytic stomatitis, mucocutaneous pyoderma, pyogranulomatous inflammation, sialadenitis, and ulcerative stomatitis (Figure 4a,b). For the remaining non-neoplastic lesions, we obtained a lower number of cytological diagnoses compared to the histological diagnoses only in granulomatous lymphadenitis (Figure 2).

When comparing the cytological and histological diagnoses of non-neoplastic oral lesions in cats, we obtained the same diagnosis for eosinophilic ulcerative stomatitis, leishmania granuloma, lymphoplasmacytic gingivitis, lymphoplasmacytic glossitis, lymphoplasmacytic stomatitis, osteomyelitis, and pyogranulomatous inflammation. For the remaining non-neoplastic lesions, we obtained a lower number of cytological diagnoses compared to the histological diagnoses: granulation tissue-type hemangiomas and suppurative inflammation. The values obtained for the previous data are shown in Figure 2.

When we compared the cytological and histological diagnoses of neoplastic and non-neoplastic, we obtained the same diagnosis in 64.0% (*n* = 66) and 23.3% (*n* = 24), respectively; for the remaining diagnoses, there was no diagnostic coincidence, as can be seen in Table 3.

A chi-square test was performed for the neoplastic and non-neoplastic lesions diagnosed by cytology and diagnosed by histopathology. When we evaluated this association, we obtained a significant *p*-value (*p* < 0.0001).

A chi-square test was performed for the neoplastic and non-neoplastic lesions and their level of agreement, with the data presented in Table 4. When we evaluated the association between the neoplastic cases and agreement, we obtained a significant *p*-value (*p* < 0.0001). On the other hand, the same association for non-neoplastic cases revealed a non-significant *p*-value (*p* = 0.3173).

### 3.4. Level of Agreement and Diagnostic Accuracy

In the present study, we obtained a sensitivity of 84.6%, a specificity of 96.0%, and an accuracy of 87.4% in the cytological diagnosis.

Of the overall 103 cases, 75.7% (*n* = 78) were neoplastic and revealed a diagnosis agreement that was complete in 64.1% (*n* = 50) and partial in 17.9% (*n* = 14) of the samples, and the remaining 17.9% (*n* = 14) revealed disagreement; for the non-neoplastic diagnoses (24.3%, *n* = 25), the agreement was complete in 68.0% (*n* = 17) and partial for 20.0% (*n* = 5) samples, and the remained 12.0% (*n* = 3) revealed disagreement (Table 4).

Concerning the 70 dogs, 84.3% (*n* = 59) of cases were neoplastic and revealed a diagnosis agreement that was complete in 66.1% (*n* = 39) and partial in 16.9% (*n* = 10), and the remaining 16.9% (*n* = 10) revealed disagreement; regarding the non-neoplastic diagnoses (15.6%, *n* = 11), the agreement was complete in 72.7% (*n* = 8) and partial for 18.1% (*n* = 2), and the remaining 9.1% (*n* = 1) revealed disagreement (Table 4).

Concerning the 33 cats, 57.6% (*n* = 19) of cases were neoplastic and revealed a diagnoses agreement that was complete in 57.9% (*n* = 11) and partial in 21.1% (*n* = 4), and the remaining 21.1% (*n* = 4) revealed disagreement; regarding the non-neoplastic diagnoses (42.4%, *n* = 14), the agreement was complete in 64.3% (*n* = 9) and partial for 21.4% (*n* = 3), and the remaining 14.3% (*n* = 2) revealed disagreement (Table 4).

In 14 cases with a histological diagnosis of neoplasia, the previous cytological diagnosis indicated another lesion; therefore, they were classified as disagreement cases. One case of melanoma was diagnosed as an abscess; one case of squamous cell carcinoma as a benign hair follicular tumor; two lymphomas as reactive lymph node hyperplasia and chronic granulomatous inflammation; three cases of peripheral odontogenic fibroma as pyogranulomatous inflammation, septic suppurative inflammation, and a discrete cell tumor; three salivary gland adenocarcinomas as two abscesses and one pyogranulomatous inflammation; one case of mast cell tumor as a hematoma; two cases of undifferentiated malignancy as a septic suppurative inflammation and bacterial stomatitis; and one histiocytoma as a lymphoplasmacytic inflammation (Table 5 and Table S1).

There were also two cases diagnosed histologically as neoplasms—squamous cell carcinoma and peripheral odontogenic fibroma, whose cytological diagnosis was also neoplasia, although the cell type and the classification of the type of tumor was discordant—benign hair follicular tumor and discrete cell tumor, respectively (Table 5).

Concerning the non-neoplastic lesions, in three cases with a histological diagnosis of different kind of processes (granulation tissue-type hemangiomas, lymphoplasmacytic stomatitis, and granulomatous lymphadenitis), the previous cytological diagnosis indicated another type of inflammatory lesion—abscess, suppurative inflammation with dysplastic epithelial cells, and reactive lymph node hyperplasia, respectively. Therefore, they were classified as disagreement cases (Table 6 and Table S2).

The statistical analysis, regarding the agreement, found that it was not related to the location of the lesions (*p* = 0.72). However, a significant association was observed between the neoplastic and non-neoplastic groups diagnosed by cytology and diagnosed by histology (*p* < 0.0001), which means that the cytological and histopathological diagnoses were related. A significant association was observed between the neoplastic group and the level of agreement (*p* < 0.0001); otherwise, when considering the non-neoplastic group, the statistical association was not significant (*p* = 0.3173). There are more neoplastic cases in the agreement and partially agreement groups, unlike the disagreement group where non-neoplastic lesions appeared in a larger amount; these data are presented in Table 4.

## 4. Discussion

In the present study, we determined the agreement between cytological and histopathological diagnoses in tumor and tumor-like samples from canine and feline oral lesions. Cytology is a minimally invasive, rapid, and cheap diagnostic tool, routinely used in small animal clinical practice [1,2,30,33,34,36]. Throughout the years, fine-needle aspiration cytology has been found to be very valuable in evaluating and diagnosing various neoplastic and non-neoplastic lesions of many anatomical locations, namely in the oral cavity [22]. For masses, this technique allows the aspiration of cellular content from deep layers, avoiding superficial inflammation and debris, being more rewarding and avoiding more invasive procedures such as histopathological biopsies [43]. For these reasons, it is highly used in the clinical context and also strongly recommended, particularly in space-occupying lesions [1,33,35,44]. Despite these benefits, some weaknesses exist, such as the lack of tissue architecture and the fact that it is not always possible to obtain samples with good cellularity, either because of sampling difficulties or because of the tissue characteristics as, for example, in mesenchymatous lesions due to their low exfoliative capacity [15,19,20]. In the context of clinical practice, the most important feature for the clinician is being able to distinguish between neoplastic and non-neoplastic processes [1]. In the specific case of the oral cavity, some samples may not be easy to collect, either due to deeply located intra-oral locations, making it difficult to hold the lesion and handle the needle, or due to difficulties in handling nervous animals [22]. On the other hand, associated with this difficulty is the use of less appropriate collection techniques, such as the apposition technique (direct touch imprint) in ulcerated oral masses, which may contribute to an increase in false negatives since this method will only allow the collection of cells from the most superficial layers, often contaminated by inflammation, microorganisms, blood, and cellular debris [45].

Explanations for the non-concordance of diagnoses between the cytological and histological samples may be related to poor sample collection or inadequate collection technique, cytological collection not being performed at the same site as the biopsy, and the time elapsed between the cytological and histological collection being extensive and allowing for the alteration or progression of the underlying process [10]. Additionally, the clinician’s experience in performing the collection and their selection of the area of interest cannot be underestimated, because they are intrinsically related to the cellularity and quality of the sample [2,5,26].

Even in studies in which the operators performing the cytology were the same, from three large clinical centers and with a high level of experience, as in the study by Bonfanti et al. (2015) [2], 14% of the cases were excluded because the cytological results were unsatisfactory with all cytological sampling methods. In our study, the number of excluded cases was higher, 25.4%, which may have occurred due to the multiple collectors from different clinics and with various level of experience, which seems to influence the rate of samples with material and eventually affects the concordance.

In fact, in oral lesions, only one study exists [2] regarding the diagnostic value of cytological analysis in dogs and cats. Our study was conducted retrospectively, and the method of cytological achievement was not controlled, which means that all the sampling was conducted onsite by clinicians with different training and expertise. On the other hand, in Bonfanti et al.’s (2015) [2] study a prospective approach was made using and comparing three different techniques of sample collection—fine-needle aspiration, fine-needle insertion, the non-aspiration technique, and impression smears. While most of our samples were collected by fine-needle aspiration, this information was not available in our case; so, a direct comparison with the results provided by Bonfanti et al. (2015) [2] is difficult. Still, it should be noted that the studies carried out on human oral cytological samples, in which cytology is compared with histopathology, have emphasized the benefits of the aspirative collection method [22,25].

When comparing cytological and histological diagnoses in neoplastic lesions in dogs, more than 80% of the cytological samples showed a diagnosis in agreement with the histology, and in the case of cats, around 79% were equally in agreement. In the case of dogs, the diagnoses that resulted in a higher discrepancy between the cytology and histology were peripheral odontogenic fibroma and undifferentiated malignancy, possibly justified by their predominantly mesenchymal population and, therefore, with characteristics of poor exfoliation. In the case of cats, the most discrepant diagnosis was salivary gland adenocarcinoma, justified by cell sampling from inflammatory areas, without representation of neoplastic areas (Figure 4).

When comparing the cytological and histological diagnoses in non-neoplastic lesions in dogs and cats, the concordance was higher (92% and 85%, respectively). The discrepancies were only found in inflammatory diagnoses, and this fact was probably justified by the samples being taken in areas of heterogeneous cellular distribution of the inflammatory population (Figure 4).

Some of the possible causes that may explain the diagnostic discrepancies detailed in Table 5 and Table 6 were the superficial collections carried out by apposition cytology or the collection of material from inflammatory areas (e.g., melanoma vs. abscess or septic suppurative inflammation) or hemorrhage (mast cell tumor vs. hematoma) without tumor representation; borderline situations of lesions that present transition areas between reactive and neoplastic (lymphoma vs. reactive lymph node hyperplasia) and also cases of neoplasm with an intra-lesional lymphocytic population, such as the case of histiocytomas in regression; oral lesions characterized by abundant fibrous tissue such as peripheral odontogenic fibroma that exfoliate few cells and are related with poor cellularity cytological samples; different aspiration sites of the lesion or an uneven distribution of the different inflammatory cell types within the lesion, the time elapsed between the cytological and histological sample collection and the evolution of the inflammatory process itself.

Analyzing existing studies in which cytology was correlated with histopathology, our sensitivity values were often lower, which could lead to a failure in the cytological identification of processes; however, on the other hand, the fact that there was a high specificity assures us that our cases diagnosed as negative are in fact true negatives. Some of the studies [22,27,46] showed very low sensitivities, especially in the case of cats’ lymph nodes [11], suggesting that cytology may not be as useful as a screening tool. However, in these cases, it could be important to use cytology as the first approach in the diagnostic process, which would exclude negative cases, followed by histopathological examination to obtain a definitive diagnosis.

A literature review on the concordance between cytological and histopathological diagnoses on lesions located in the oral cavity [2] and in other tissues is presented in Table 7 [1,21,22,23,27,29,30,31,38,42,46,47]. For the sake of illustration, two studies in the oral cavity of humans were also included [22,25].

In our study, we obtained a sensitivity of 84.6%, a specificity of 96.0%, and an accuracy of 87.4%, considering the total of samples assessed without sorting by species. When comparing our results with two human studies—Khan et al. (2013) [33], performed on 199 oral cytology samples, and Singhal et al. (2015) [36], performed on 50 oral cytology samples—we verified that their sensitivity values (93.2% and 90%) and accuracy (94.9% and 94.1%) were higher, and the specificity values (96.8% and 97.5%) were similar. Probably, the differences encountered in the sensitivity and accuracy values may be due to the existence of a high number of false negative cases in our study (*n* = 12) [22,25].

In studies carried out on various anatomical locations by Ghisleni et al. (2006) [1], Chalita et al. (2001) [23], and Sontas et al. (2011) [30] the sensitivity, specificity, and accuracy values were higher than in our study, with the exception of Simon’s work (2009), which had the same specificity as ours. This could be due to the tissue location of those studies (skin and mammary gland) that is more readily accessible and easy to collect than in the oral cavity; specifically, in Sontas et al. (2011) [30], the authors justified the values obtained with an increase in the number of aspirations performed (three or more). In Bonfanti et al.’s (2004) [42] and Eich et al.’s (2000) [5] studies performed on visceral masses, the sensitivity and specificity values obtained were slightly higher than those presented herein (Table 7).

Other studies performed on mammary, lymph nodes, lung, and bone by Allen et al. (1986) [27], Ku et al. (2017) [22], Deberry et al. (2002) [46], and Sabattini et al. (2017) [31], respectively, and in multiple locations by Cohen et al. (2003) [21] and Vos et al. (1989) [47] revealed sensitivity, specificity, and accuracy values lower than those we obtained, with the exception of the specificity in Deberry et al.’s [46] and Ku et al.’s [22] studies (feline population) and the sensitivity in Vos et al.’s study. These dissimilar results may be explained by differences in the studies’ concepts and methodologies (Table 7).

Our retrospective study permitted us to determine the association between diagnostic cytology and histopathology in neoplastic and non-neoplastic samples from oral cavity lesions of dogs and cats regardless of the collection technique used. The results obtained, together with the work already carried out by Bonfanti et al. (2015) [2] also on oral lesions in dogs and cats, may allow a global version of the impact of cytological examination on diagnoses.

## 5. Conclusions

Our work could be helpful for both pathologists and clinicians in understanding the diversity and challenges of diagnosing oral lesions, both neoplastic and inflammatory; in addition, the high association verified in our study between cytological diagnosis and the definitive diagnosis given by histopathological examination emphasize the role of cytology as the initial method of diagnosis of an oral sample. Furthermore, cytology as an inexpensive and non-invasive technique used as a first-line approach can contribute to the diagnosis of the lesions, namely neoplastic at an early stage, thus contributing to more appropriate, prompt, and specific therapy for each animal.

## Figures and Tables

**Table 1:** Location of the oral lesions in dogs and cats.

Location	Dogs	Cats	Total
	*n* (%)	*n* (%)	*n* (%)
**Gingiva**	17 (24.3)	5 (15.2)	22 (21.4)
**Mouth not otherwise specified (MNOS)**	15 (21.4)	7 (21.2)	22 (21.4)
**Lips**	17 (24.3)	3 (9.1)	20 (19.4)
**Tongue**	2 (2.9)	9 (27.3)	11 (10.7)
**Palate**	5 (7.1)	2 (6.1)	7 (6.8)
**Jaw**	4 (5.7)	2 (6.1)	6 (5.8)
**Submandibular lymph nodes**	4 (5.7)	0 (0)	4 (3.9)
**Maxilla**	2 (2.9)	1 (3.0)	3 (2.9)
**Salivary glands**	2 (2.9)	1 (3.0)	3 (2.9)
**Oropharynx**	1 (1.4)	1 (3.0)	2 (1.9)
**Tonsils**	1 (1.4)	1 (3.0)	2 (1.9)
**Dental**	0 (0.0)	1 (3.0)	1 (1.0)
**Total**	70 (100)	33 (100)	103 (100)

**Table 2:** Analysis of the level of agreement between the cytological and histologic diagnoses with the location of oral lesions in both cats and dogs.

Total Count	Gingiva *n* (%)	Lip *n* (%)	MNOS * n (%)	Tongue *n* (%)	Total *n* (%)
**Disagreement**	5 (38.5)	3 (23.1)	3 (23.1)	2 (15.4)	13 (100)
**Agreement**	12 (23.1)	15 (28.8)	17 (32.7)	8 (15.4)	52 (100)
**Partial Agreement**	5 (50.0)	2 (20.0)	2 (20.0)	1 (10.0)	10 (100)

* MNOS—Mouth not otherwise specified.

**Table 3:** Analysis of the relation between neoplastic and non-neoplastic lesions diagnosed by cytology and diagnosed by histopathology.

Count Total%	Neoplastic Histopathology *n* (%)	Non-Neoplastic Histopathology *n* (%)	Total *n* (%)
**Neoplastic** **cytology**	66 (64.0)	1 (1.0)	67 (65.0)
**Non-neoplastic** **cytology**	12 (11.7)	24 (23.3)	36 (35.0)
**Total** ***n* (%)**	78 (75.7)	25 (24.3)	103 (100)

**Table 4:** Analysis of the level of agreement in the two diagnosis groups (neoplastic vs. non-neoplastic).

	Concordance	Neoplastic *n* (%)	Non-Neoplastic *n* (%)	Total *n* (%)
**Global** **Concordance**	Agreement	50 (64.1)	17 (68.0)	67 (65.0)
	Partial Agreement	14 (17.9)	5 (20.0)	19 (18.5)
	Disagreement	14 (17.9)	3 (12.0)	17 (16.5)
**Total Concordance**		78 (75.7)	25 (24.3)	103 (100)
**Dogs**	Agreement	39 (66.1)	8 (72.7)	47 (67.2)
	Partial Agreement	10 (16.9)	2 (18.1)	12 (17.1)
	Disagreement	10 (16.9)	1 (9.1)	11 (15.7)
**Total Dogs**		59 (84.3)	11 (15.6)	70 (100)
**Cats**	Agreement	11 (57.9)	9 (64.3)	20 (60.6)
	Partial Agreement	4 (21.1)	3 (21.4)	7 (21.2)
	Disagreement	4 (21.1)	2 (14.3)	6 (18.2)
**Total Cats**		19 (57.6)	14 (42.4)	33 (100)
**Total Dogs/Cats**		78 (75.7)	25 (24.3)	103 (100)

**Table 5:** Analysis of agreement, partial agreement, and disagreement in neoplasia diagnoses.

Neoplastic Lesions	Number of Cases *n* (%)	Agreement	Partial Agreement	Disagreement	
				Number of Cases *n*	Cytological Diagnosis
**Melanoma**	15 (19.2)	10	4	1	Abscess
**Squamous cell carcinoma**	18 (23.1)	13	4	1	Benign hair follicle tumor
**Lymphoma**	8 (10.3)	6	0	2	Reactive lymph node hyperplasia Chronic granulomatous inflammation
**Fibrosarcoma**	7 (9.0)	7	0	0	-
**Peripheral odontogenic fibroma**	6 (7.7)	3	0	3	Pyogranulomatous inflammation Discrete cell tumors Septic suppurative inflammation
**Salivary gland adenocarcinoma**	5 (6.4)	1	1	3	Abscess (2) Pyogranulomatous inflammation
**Mast cell tumor**	4 (5.1)	3	0	1	Hematoma
**Undifferentiated Malignancy**	6 (7.7)	3	1	2	Septic suppurative inflammation Bacterial stomatitis
**Osteosarcoma**	1 (1.3)	1	0	0	-
**Chondrosarcoma**	1 (1.3)	1	0	0	-
**Histiocytic sarcoma**	1 (1.3)	1	0	0	-
**Hepatoid gland epithelioma**	1 (1.3)	0	1	0	-
**Sebaceous epithelioma**	1 (1.3)	0	1	0	-
**Histiocytoma**	1 (1.3)	0	0	1	Lymphoplasmacytic inflammation
**Papilloma**	1 (1.3)	0	1	0	-
**Trichoblastoma**	1 (1.3)	1	0	0	-
**Apocrine gland carcinoma**	1 (1.3)	0	1	0	-
**Total**	78 (100)	50	14	14	

**Table 6:** Analysis of agreement, partial agreement, and disagreement in non-neoplastic diagnoses.

Non-Neoplastic Lesions	Number of Cases *n* (%)	Agreement	Partial Agreement	Disagreement	
				Number of Cases *n*	Cytological Diagnosis
**Granulation tissue-type hemangiomas**	**3 (12.0)**	**1**	**1**	**1**	Abscess
**Lymphoplasmacytic stomatitis**	**3 (12.0)**	**0**	**2**	**1**	Suppurative inflammation with dysplastic epithelial cells
**Suppurative inflammation**	**2 (8.0)**	**2**	**0**	**0**	-
**Sialoadenitis**	**2 (8.0)**	**2**	**0**	**0**	-
**Ulcerative stomatitis**	**2 (8.0)**	**2**	**0**	**0**	-
**Pyogranulomatous inflammation**	**2 (8.0)**	**2**	**0**	**0**	-
**Abscess**	**1 (4.0)**	**1**	**0**	**0**	-
**Eosinophilic ulcerative stomatitis**	**1 (4.0)**	**1**	**0**	**0**	-
**Granulomatous gingivitis**	**1 (4.0)**	**1**	**0**	**0**	-
**Lymphoplasmacytic gingivitis**	**1 (4.0)**	**1**	**0**	**0**	-
**Eosinophilic granuloma**	**1 (4.0)**	**0**	**1**	**0**	-
**Granulomatous inflammation**	**1 (4.0)**	**1**	**0**	**0**	-
**Lymphoplasmacytic glossitis**	**1 (4.0)**	**0**	**1**	**0**	-
**Granulomatous lymphadenitis**	**1 (4.0)**	**0**	**0**	**1**	Reactive lymph node hyperplasia
**Leishmania granuloma**	**1 (4.0)**	**1**	**0**	**0**	-
**Mucocutaneous pyoderma**	**1 (4.0)**	**1**	**0**	**0**	-
**Osteomyelitis**	**1 (4.0)**	**1**	**0**	**0**	-
**Total**	**25 (100)**	**17**	**5**	**3**	-

**Table 7:** Compilation of the published veterinary and human studies about the association between cytological and histopathological diagnoses.

Studies	Species	Location	Number of Cases	Sensitivity	Specificity	Accuracy
**Current study**	Dogs and cats	Oral	103	84.6%	96.0%	87.4%
**Bonfanti et al., 2015**	Dogs	Oral	96	FNA (100%); FNI (98%); IS (91.1%)	FNA (75%); FNI (100%); IS (100%)	FNA (98.2%); FNI (98.1%); IS (91.8%)
	Cats			FNA (94.7%); FNI (94.4%); IS (94.1%)	FNA (100%); FNI (100%); IS (100%)	FNA (95.6%); FNI (95.6%); IS (95.8%)
**Khan et al., 2013**	Human	Oral	199	93.2%	96.8%	94.9%
**Singhal et al., 2015**	Human	Oral	50	90%	97.5%	94.1%
**Simon et al., 2009**	Dogs	Mammary	50	88%	96%	93%
**Allen et al., 1986**	Dogs	Mammary	91	Cytologist 1 (25%); Cytologist 2 (17%)	Cytologist 1 (62%); Cytologist 2 (49%)	Cytologist 1 (79%); Cytologist 2 (76%)
**Sontas et al., 2011**	Dogs	Mammary	90	96.2%	100%	96.5%
**Ku et al.,** **2016**	Dogs	Lymph nodes	367	73.3%	89.5%	80.2%
	Cats			39%	100%	64.7%
**Ghisleni et al., 2006**	Dogs and cats	Skin masses	243	89.3%	97.9%	-
**Chalita et al., 2001**	Dogs	Skin and soft tissue	85	89%	100%	97%
**Cohen et al., 2003**	Dogs, cats, horses, ferrets, llamas, rats, and mice	Multiple	269	63.2%	82.5%	-
**Vos et al.,** **1989**	Dogs	Multiple, including oral	322	95.6%	65.4%	83.9%
**Bonfanti et al., 2004**	Dogs and cats	Deep thoracic and abdominal masses	132	87.8%	100%	89.4%
**Eich et al.,** **2000**	Dogs, cats, and exotic animals	Masses of various organ systems	100	89%	100%	87%
**Deberry et al., 2002**	Dogs and cats	Lung	28	77%	100%	82%
**Sabattini et al., 2017**	Dogs	Bone	68	83.3%	80%	83%

## Data Availability

The data may be requested from the corresponding author.

## Supplementary Materials

The following supporting information can be downloaded at: https://www.mdpi.com/article/10.3390/vetsci12020075/s1, Table S1: Analysis of agreement, partial agreement and disagreement in neoplasia diagnoses; Table S2: Analysis of agreement, partial agreement and disagreement in non-neoplastic diagnoses.
