# Supplementary material for: Association Between Cytological and Histopathological Diagnoses of Neoplastic and Non-Neoplastic Lesions in Oral Cavity from Dogs and Cats: An Observational Retrospective Study of 103 Cases
Source: Vet Sci. 2025 Jan 21;12(2):75. doi: 10.3390/vetsci12020075 (PMC11861943; doi:10.3390/vetsci12020075)
Supplement: Supplementary file 1 [file vetsci-12-00075-s001.zip › vetsci-3296619-supplementary.pdf]

Table S1. Analysis of agreement, partial agreement and disagreement in neoplasia diagnoses.

Neoplastic Lesions	Number of cases *n* (%)	Agreement	Partial Agreement		Disagreement	
			Number of cases *n*	Cytological diagnosis	Number of cases *n*	Cytological diagnosis
**Melanoma**	15 (19.2)	10	4	- Inflamed mesenchymal neoplasia<br>- Mesenchymal neoplasia with pyogranulomatous inflammation<br>- Discrete (round) cell tumor, slightly pigmented<br>- Fibrosarcoma	1	Abscess
**Squamous cell carcinoma**	18 (23.1)	13	4	- Epithelial hyperplasia/dysplasia with inflammation (3)<br>-Basal cell tumor	1	Benign hair follicle tumor
**Lymphoma**	8 (10.3)	6	0	-	2	Reactive lymph node hyperplasia<br>Chronic granulomatous inflammation
**Fibrosarcoma**	7 (9.0)	7	0	-	0	-
**Peripheral odontogenic fibroma**	6 (7.7)	3	0	-	3	Pyogranulomatous inflammation<br>Discrete cell tumors<br>Septic suppurative inflammation
**Salivary gland adenocarcinoma**	5 (6.4)	1	1	Basal cell tumor	3	Abscess (2)<br>Pyogranulomatous inflammation
**Mast cell tumor**	4 (5.1)	3	0	-	1	Hematoma
**Undifferentiated malignancy**	6 (7.7)	3	1	Granulation tissue	2	Septic suppurative inflammation<br>Bacterial stomatitis
**Osteosarcoma**	1 (1.3)	1	0	-	0	-

**Chondrosarcoma**	1 (1.3)	1	0	-	0	-
**Histiocytic sarcoma**	1 (1.3)	1	0	-	0	-
**Hepatoid gland epithelioma**	1 (1.3)	0	1	Carcinoma	0	-
**Sebaceous epithelioma**	1 (1.3)	0	1	Trichoblastoma	0	-
**Histiocytoma**	1 (1.3)	0	0	-	1	Lymphoplasmacytic inflammation
**Papilloma**	1 (1.3)	0	1	- Epithelial hyperplasia/dysplasia with inflammation	0	-
**Trichoblastoma**	1 (1.3)	1	0	-	0	-
**Apocrine gland carcinoma**	1 (1.3)	0	1	- Basal cell tumor	0	-
**Total**	78 (100)	50	14		14	

Table S2. Analysis of agreement, partial agreement and disagreement in non-neoplastic diagnoses.

**Non-Neoplastic Lesions**	**Number of cases *n* (%)**	**Agreement**	**Partial Agreement**		**Disagreement**	
			**Number of cases *n***	**Cytological diagnosis**	**Number of cases *n***	**Cytological diagnosis**
**Granulation tissue-type hemangiomas**	**3 (12.0)**	**1**	**1**	- Granulomatous inflammation	**1**	Abscess
**Lymphoplasmacytic stomatitis**	**3 (12.0)**	**0**	**2**	- Chronic suppurative inflammation<br>- Mixed inflammation	**1**	Suppurative inflammation with dysplastic epithelial cells
**Suppurative inflammation**	**2 (8.0)**	**2**	**0**	**-**	**0**	-
**Sialoadenitis**	**2 (8.0)**	**2**	**0**	**-**	**0**	-
**Ulcerative stomatitis**	**2 (8.0)**	**2**	**0**	**-**	**0**	-
**Pyogranulomatous inflammation**	**2 (8.0)**	**2**	**0**	**-**	**0**	-

**Abscess**	**1 (4.0)**	**1**	**0**	**-**	**0**	-
**Eosinophilic ulcerative stomatitis**	**1 (4.0)**	**1**	**0**	**-**	**0**	-
**Granulomatous gingivitis**	**1 (4.0)**	**1**	**0**	**-**	**0**	-
**Lymphoplasmacytic gingivitis**	**1 (4.0)**	**1**	**0**	**-**	**0**	-
**Eosinophilic granuloma**	**1 (4.0)**	**0**	**1**	- Mixed inflammation	**0**	-
**Granulomatous inflammation**	**1 (4.0)**	**1**	**0**	**-**	**0**	-
**Lymphoplasmacytic glossitis**	**1 (4.0)**	**0**	**1**	- Chronic suppurative inflammation	**0**	-
**Granulomatous lymphadenitis**	**1 (4.0)**	**0**	**0**	**-**	**1**	Reactive lymph node hyperplasia
**Leishmania granuloma**	**1 (4.0)**	**1**	**0**	**-**	**0**	-
**Mucocutaneous pyoderma**	**1 (4.0)**	**1**	**0**	**-**	**0**	**-**
**Osteomyelitis**	**1 (4.0)**	**1**	**0**	**-**	**0**	-
**Total**	**25 (100)**	**17**	**5**		**3**	
